# Functional disorders of the lung and symptoms of respiratory disease associated with occupational inhalation exposure to wood dust in Iran

**DOI:** 10.4178/epih.e2018031

**Published:** 2018-07-04

**Authors:** Masoud Neghab, Zeinab Jabari, Fatemeh Kargar Shouroki

**Affiliations:** 1 Research Center for Health Sciences, Institute of Health, Shiraz University of Medical Sciences, Shiraz, Iran; 2 Student Research Committee, Shiraz University of Medical Sciences, Shiraz, Iran

**Keywords:** Wood, Dust, Fungi, Bacteria, Respiratory function tests, Iran

## Abstract

**OBJECTIVES:**

The possible adverse respiratory effects of airborne pollutants in sawmills have not been thoroughly investigated in Iran. Additionally, the extent to which workers are exposed to this organic dust and its associated bioaerosols has not been extensively quantified. Likewise, the predominant bacterial and fungal species associated with wood dust have not been characterized. The present study was undertaken to address these issues.

**METHODS:**

One hundred male individuals exposed to wood dust and 100 unexposed male subjects were investigated. They completed a standardized respiratory symptom questionnaire and underwent spirometry testing. Additionally, airborne concentrations of respirable and inhalable dust particles, bacteria, and fungi were measured.

**RESULTS:**

The mean concentrations of inhalable and respirable dust particles, bacteria, and fungi were found to be 2.44, 6.76 mg/m^3^ , 756.38, and 299.15 colony-forming units/m^3^ , respectively. The predominant Gram-negative bacteria in the sawmills included the Pseudomonadaceae, *Klebsiella pneumoniae*, and *Rhinoscleromatis* spp., and the predominant fungi consisted of the zygomycetes and *Aspergillus* spp. Respiratory symptoms were significantly more prevalent among exposed workers. Significant cross-shift decrements were noted in some pulmonary function parameters. Similarly, pre-shift spirometry results indicated that some pulmonary function parameters were significantly lower in the exposed group.

**CONCLUSIONS:**

Exposure to wood dust and its bioaerosols was associated with significantly higher prevalence of respiratory symptoms and both acute (i.e., partially reversible) and chronic (i.e., irreversible) decrements in the functional capacity of the lung. Additionally, the characterized bioaerosols did not differ significantly from those isolated in other parts of the world.

## INTRODUCTION

Wood is one of the most important renewable natural resources in the world [1]. It is used in many processing industries, such as chipboard manufacturing, woodworking, furniture making, or on a smaller scale in enterprises such as carpentry [2]. Wood dust is one of the most common sources of occupational dust exposure [3]. It is estimated that 1,700 million m^3^ of forests are harvested for industrial purposes annually, and at least 2 million people per day are exposed to wood dust [1]. Wood dust is a complex mixture generated when wood is cut and shaped through processes such as grinding, sawing, turning, drilling, and grinding [1]. The American Conference of Governmental Industrial Hygienists has set an 8-hour threshold limit value (TLV) of 1 mg/m^3^ for certain hardwoods such as beech and oak and 5 mg/m^3^ for soft wood [4].

Studies have shown that wood dust mainly includes particles larger than 10 microns [5]. Respiratory diseases are the most common occupational diseases. Pulmonary function tests (PFTs), including spirometry, play an essential role in diagnosing pulmonary diseases [6]. Wood dust is heavily contaminated with fungi and Gram-negative bacteria, especially in hot and humid areas. Occupational inhalation exposure to wood dust and its associated bioaerosols has been associated with adverse respiratory effects [7-11]. Endotoxins, Gram-negative bacteria, fungi, and β-d-(1-3)-glucan are potential biological agents found in organic dust such as wood dust. Exposure to these agents can result in organic dust toxic syndrome, upper respiratory tract infections, and extrinsic allergic alveolitis [7,12,13]. Some studies have shown that inhalation of wood dust can lead to occupational asthma [11,14,15], irritation of the eyes or nose [15-17], dermatitis [18,19], nasal cancer [20-22], and respiratory tract cancers [23,24].

Previous studies have shown a significant association between inhalation of wood dust and an increased prevalence of respiratory symptoms [2,17,25-27] and decreased lung functional capacity, as manifested by reduced peak expiratory flow (PEF), forced vital capacity (FVC), and FEV1 stands for forced expiratory volume in the first second [2,7,17,25-29]. For instance, Osman & Pala [29] showed that occupational exposure to wood dust was associated with nasal congestion (53%), redness of the eyes (43%), itchy eyes (41%), and rhinorrhea (23%), in addition to decreased FVC and FEV1 values. The results of Mandryk et al. [7] also showed that exposure to endotoxin, β-d-glucan, Gram-negative bacteria, and fungi in workers at a green mill was significantly higher than in workers at a dry mill. Comparison of these 2 groups revealed a higher exposure-related prevalence of regular cough, nasal congestion, sneeze, sinus problems, flu-like symptoms, and eye and throat irritation. Conversely, some other studies have not shown any significant association between occupational exposure to wood dust and pulmonary function parameters [30-32]. For example, in a study by Liou et al. [30], the FEV1/FVC ratios of the exposed and reference groups were not significantly different. Additionally, the prevalence of respiratory symptoms in the exposed group, including smokers and non-smokers, was not significantly higher than in the reference group.

In light of the above findings, controversy exists regarding the potential of wood dust to produce respiratory symptoms or functional abnormalities in the lungs. Additionally, despite the fact that exposure to wood dust, due to its wide applications in different industries, is pervasive, there is a paucity of studies in which the respiratory effects of this organic dust have been evaluated at the national or regional level. Finally, to the best of our knowledge, bioaerosols contaminating wood dust have not previously been characterized and quantified in Iran. This study was undertaken to more thoroughly investigate and address these issues.

## MATERIALS AND METHODS

This cross-sectional study was carried out among all employees (100 male workers exposed to airborne pollutants) of 20 randomly selected sawmills (5 employees in each sawmill, on average) in Shiraz and 100 randomly selected male appliance retailers working in nearby plants, adjacent to the sawmills, as the reference group. The exposed group was comparable to the reference group in terms of sample size, age, sex, smoking habits, and other variables such as marital status, income, and education. Additionally, they had no history of occupational and non-occupational exposure to dust or other chemicals with known pulmonotoxic properties, and were free from pre-existing medical conditions such as asthma and chronic respiratory diseases or a history of thoracic surgery. The subjects in the exposed group were more or less similar in terms of their exposure to wood dust and bioaerosols, and they worked 8-hour daily shifts (8 a.m. to 4 p.m.). Each participant completed a questionnaire to ensure that they met the inclusion criteria.

### Sampling for dust measurements

The National Institute for Occupational Safety and Health method (NIOSH) method 0500 [33] was used to quantify workers’ exposure to wood dust. The concentrations of respirable (< 5 µm) and inhalable dust particles (> 5 µm) were measured by a personal dust sampler equipped with a 0.5 micron (pore size) polyvinyl chloride filter attached to a cyclone (SKC Ltd., Dorset, UK). Pretest measurements showed that the appropriate flow rate and sampling time to avoid filter overloading were 2 liters per minute and 60 minutes, respectively. The filters and the contents of the cyclone were weighed with a Sartorius balance (Karl-ArnoldStraße 2137079, Goettingen, Germany) with a sensitivity of 0.0001 g before and after sampling, and then workers’ exposure to wood dust was calculated.

#### Bioaerosol sampling

Using NIOSH method 0800 [34], bacteria and fungi were sampled by an Andersen single-stage sampler with a flow rate of 28.3 L/min. The culture media was malt extract agar for airborne fungi and trypticase soy agar for bacteria. The sampling time was 10 minutes. To calculate the concentration of colonies in the culture medium, the air volume was corrected relative to the air pressure and temperature during sampling, and the bioaerosol concentration was calculated in terms of colony-forming units (CFU)/m^3^ . Finally, microbiological and mycological tests were performed on the plates. An API 20E microbial identification kit (bioMerieux Inc., Durham, NC, USA) was used to identify the bacterial species. None of the sawmills were equipped with local exhaust ventilation systems and none of the exposed workers wore any respiratory protective equipment.

#### Standard respiratory symptom questionnaire

A valid and reliable European Community Respiratory Health Survey questionnaire [35] was used to investigate the prevalence of respiratory symptoms in the exposed and reference groups. The Cronbach alpha of this questionnaire was calculated to be 0.77 in the present study. This questionnaire included questions about respiratory symptoms (cough, phlegm, wheezing, dyspnea, etc.), smoking habits, the medical and family history of subjects, and so on.

#### Spirometry

Pulmonary function parameters, including vital capacity (VC), FVC, FEV1, PEF, and the FEV1/FVC and FEV1/VC ratios, were measured by an experienced, skillful operator (one of the authors). The test was carried out twice in the workers exposed at their workplace: (1) Prior to the shift, on the first work day of the week after an exposure-free period of at least 48 hours; (2) Immediately after the end of the daily work shift on the first work day of the week.

First, the workers were provided with the necessary training for performing spirometry. They were then asked to sit in a chair for 5 minutes to rest, and then the maneuver was conducted at least 3 times for each subject. The workers were asked to refrain from smoking and bathing at least 2 hours before spirometry [36].

### Data analysis

The data were analyzed using SPSS version 21 (IBM Corp., Armonk, NY, USA). The Student *t*-test, paired *t*-test, chi-square test, or the Fisher exact test was used for statistical comparisons, as appropriate. Linear and logistic regression analyses (backward elimination method) were used to investigate the associations of exposure to airborne contaminants with changes in pulmonary function parameters and the prevalence of respiratory symptoms, while potential confounders were controlled in the model.

To differentiate between the chronic and acute respiratory effects of wood dust, pulmonary function parameters were measured prior to the shift (following a 48-hour exposure-free period) and at the end of the shift.

Before-shift parameters that were significantly lower in exposed workers than the corresponding baseline values for their non-exposed counterparts were considered to indicate a chronic irreversible effect. In contrast, pulmonary function parameters that were significantly lower at the end of the shift than before the shift were considered to indicate an acute cross-shift decrement.

## RESULTS

As shown in Table 1, there were no statistically significant differences between the exposed and reference groups in age, height, weight, marital status, work history, mean duration of smoking, or the number of cigarettes smoked per day.

The proportion of smokers in the exposed and reference groups was 41.0 and 38.0%, respectively, which was not a statistically significant difference (p= 0.66).

However, the proportion of educated individuals in the unexposed group was significantly higher than in their exposed counterparts.

The concentrations of inhalable and respirable dust were found to be 2.44 and 6.76 mg/m^3^ , respectively. The bacterial and fungal concentrations were measured to be 756.38 and 299.15 CFU/m^3^ , respectively.

The predominant bacteria found in the present study were Gram-negative. *Klebsiella pneumonia*, *Rhinoscleromatis* spp., and Pseudomonadaceae were the predominant species/genera or families of Gram-negative bacteria found in the sawmills, in addition to Gram-positive bacteria (*Bacillus* spp.). Similarly, for fungi, zygomycetes (*Rhizopus*, *Mucor*, and *Syncephalastrum*) and *Aspergillus* spp. (*A. niger, A. fumigatus, A. flavus, A. terreus*), as well as *Penicillium* and *Fusarium* species, were identified as the predominant fungal species, genera, and classes.

The prevalence of respiratory symptoms in the 2 groups is presented in Table 2.

As can be seen, the prevalence of all symptoms in the exposed group was higher than in the non-exposed group. The differences in wheezing, chest tightness, cough, chronic cough, phlegm, and dyspnea were statistically significant (p< 0.05).

Participants in the exposed group were 4.80 times more likely to develop wheezing, 2.47 times more likely to have chest tightness, and 12.57, 2.76, 4.94, 2.02, and 4.64 times more likely to suffer from cough or to complain of chronic cough, phlegm, chronic phlegm, and dyspnea than the non-exposed group, respectively.

The prevalence of respiratory symptoms among smokers was higher than among non-smokers. Moreover, all respiratory symptoms except dyspnea showed statistically significant differences between smokers and non-smokers. Furthermore, the prevalence of respiratory symptoms among exposed cigarette smokers was higher than among non-exposed cigarette smokers.

Table 3 shows the results of PFT. Comparing the mean values of lung function parameters before and after exposure showed that exposure to wood dust during a work shift significantly reduced pulmonary function parameters such as VC, FVC, and FEV1. Additionally, pre-shift spirometry results indicated that some pulmonary function parameters, such as VC, FVC, FEV1, and PEF, were significantly lower in the exposed group than in the reference group. Moreover, the differences in VC, FVC, FEV1, FEV1/VC, and PEF became more pronounced after the work shift. Additionally, the mean values of lung function parameters were compared in exposed and non-exposed cigarette smokers. The VC, FVC, FEV1, and PEF values for exposed cigarette smokers were significantly lower than those of non-exposed cigarette smokers.

Exposed subjects with a normal spirogram or with obstructive, restrictive, or mixed lung disease were examined before and after the work shift. Of the exposed subjects, 49 had a normal spirogram after the shift, while 26, 20, and 5 exposed subjects had obstructive, restrictive, or mixed lung disease, respectively. The prevalence of these complications was statistically significantly different before and after the work shift (p< 0.001).

Table 4 compares the pulmonary function parameters between smokers in both groups. As shown, most pulmonary function parameter values were significantly lower in the exposed group than in the control subjects.

To control for the effects of confounding variables, such as cigarette smoking and other tobacco product use, age, weight, height, and work experience, the results of the study were further analyzed using logistic and multiple linear regression.

Table 5 shows the associations between exposure to airborne contaminants and the prevalence of respiratory symptoms based on logistic regression analysis. As can be seen, after controlling for confounding variables, a significant association remained between exposure to airborne contaminants and the prevalence of respiratory symptoms, except for chronic phlegm (p< 0.05).

Table 6 shows the associations between exposure to airborne contaminants and changes in lung function parameters. As the results show, significant associations were found between exposure to airborne contaminants and VC, FVC, FEV1, the FEV1/ FVC ratio, and PEF, as these parameters decreased by 14.69, 10.38, 10.55, 0.14 and 8.5 units due to wood dust exposure, respectively.

## DISCUSSION

There was no significant difference in baseline variables other than educational level between the 2 groups. The average concentration of inhalable dust in the present study was 2.44 mg/m^3^ , and the concentration of respirable dust was 6.76 mg/m^3^ . These values exceeded the existing TLV for this organic dust [6]. Similarly, the average concentration of dust reported by other researchers, such as Magagnotti et al. (1.75 mg/m^3^ ) [37], Hessel et al. (1.35 mg/m^3^ ) [38], Tobin et al. (1.39 mg/m^3^ ) [25], and Osman & Pala (2.04 mg/ m^3^ ) [29] were also higher than the TLV, although the dust concentration in the present study was slightly higher than those reported in previous studies. This can be explained, at least in part, due to the fact that in the present study, the cleaning procedures involved using an air jet and dry sweeping. Additionally, the studied plants lacked any artificial ventilation system, and most of the managers and workers were illiterate and therefore were not aware of the potential health effects of wood dust.

The predominant bacteria in the present study were Gram-negative. *K. pneumonia*, *Rhinoscleromatis* spp., and Pseudomonadaceae were the dominant species/genera and classes of Gram-negative bacteria found in sawmills, in addition to Gram-positive bacteria (*Bacillus* spp.). Similarly, for fungi, zygomycetes (*Rhizopus*, *Mucor*, and *Syncephalastrum*) and *Aspergillus* spp. (*A. niger, A. fumigatus, A. flavus, A. terreus*) as well as *Penicillium* and *Fusarium* species, were identified to be the dominant fungal species, genera, and classes.

The average concentrations of bacteria and fungi were 756.38 and 299.15 CFU/m^3^ , respectively. The predominant fungi, Grampositive bacteria, and Gram-negative bacteria reported by Oppliger et al. [39] were likewise Penicillium, *Bacillus*, and Pseudomonadaceae, respectively. The bacterial and fungal concentrations in his study were 3,471 and 11,140 CFU/m^3^ , respectively. The bacterial concentration reported by Gioffrè et al. [9] was 130-2,000 CFU/m^3^ , and the predominant fungal species were *A. niger, A. flavus*, and *Penicillium* spp., in accordance with our results. In the study of Prażmo et al. [12], the dominant bacteria were *Enterobacteriaceae* and *Rahnella* spp. The dominant fungi found by Badirdast et al. [40] were *Penicillium* and *A. niger*, and the concentration of fungal aerosol was 269 CFU/m^3^ . Although the health effects of bioaerosol exposure have been identified, TLVs have not been established for them [12]. This can be explained by the diversity of bioaerosols and their different pathogenic potential. The bacterial concentration in the present study was lower than the value suggested by Clark (1,000 CFU/m^3^ ) [41]. Differences in bacterial and fungal concentrations and species between other studies and the present study can be attributed to differences in temperature and relative humidity, wood types, ventilation systems, the sampling season, and/or woodworking processes.

In this study, the prevalence of all respiratory symptoms, including wheezing (37%), cough (28%), chronic cough (15%), phlegm (24%), chronic phlegm (14%), shortness of breath (dyspnea) (74%), and chest tightness (27%), in the exposed group was higher than in the reference group, and the difference was statistically significant for all symptoms other than chronic phlegm. After controlling for confounding variables (age, height, weight, work experience, smoking, etc.) by multivariate logistic regression, a significant association remained between exposure to airborne contaminants and the prevalence of respiratory symptoms. These results are in line with those of a previous study conducted by authors on respiratory effects of exposure to bioaerosols [42]. In some studies of wood workers, the prevalence of respiratory symptoms in the exposed group was higher than that of the reference group [2, 17,25-27]. The results of those studies are consistent with the present study. In the study conducted by Bislimovska et al. [2] on 37 subjects, the prevalence of cough (29.7%) and phlegm (16.2%) in the exposed group was significantly higher than in the reference group. Moreover, according to Shamssain [28] the prevalence of all respiratory symptoms, including cough (40.6%), phlegm (24.1%), wheezing (12.8%), and dyspnea (18.0%) in the exposed group was significantly higher than in the reference group. In contrast, the results of some other studies are not consistent with the present study, as they did not report any association between exposure to airborne contaminants and the prevalence of respiratory symptoms. Similarly, those studies indicated no significant difference in the prevalence of respiratory symptoms between the exposed and non-exposed groups [30,31,43]. For instance, Borm et al. [31] in a study of 496 men with an average age of 31.0 years and 9.6 years of work experience, of whom 50% were cigarette smokers, who were exposed to wood dust concentrations of more than 5 mg/m^3^ , found no significant differences between exposure levels regarding the prevalence of respiratory symptoms. This discrepancy may be due to differences in age, work experience, humidity, dust concentration, types of fungal and bacterial contaminants, statistical analysis, confounding variables (controlled or not controlled), and/or the quality of wood dust (hard, soft, allergenic or non-allergenic).

In the present study, in order to differentiate between the acute and chronic effects of exposure to airborne contaminants in sawmills, lung function parameters were measured before and after the work shift and compared with each other and with the reference group. It is noteworthy that VC, FVC, and FEV1 were significantly different before and after the work shift. These findings indicate that exposure to wood dust during a work shift acutely reduces some parameters of pulmonary function. In addition, the differences in VC, FVC, FEV1, and PEF between the exposed group were statistically significant (before exposure on the first work day of a week after a 2-day exposure-free period) and the reference group.

The fact that PET values in the exposed workers, even after cessation of exposure and a 48-hour exposure-free period, were still significantly lower than those of the control group indicates that the exposed subjects suffered from irreversible chronic respiratory disorders. Moreover, because additional, statistically significant cross-shift decrements were noted in the PFT results, it is concluded that the exposed workers also experienced acute, partially reversible pulmonary effects.

These results are consistent with the studies of other researchers [2,11,17,27,29,44]. Mandryk et al. [45] conducted a study of 105 wood workers (with an average age of 38 years and 9 years of work experience) and concluded that the mean values of lung function parameters (VC, FVC and FEV1) were significantly lower in the exposed group. Additionally, significant cross-shift decrements in these parameters were observed. Furthermore, Mandryk et al. [7] studied a group of 87 wood workers with an average age of 36 years and 7 years of work experience, of whom 46% were smokers. They concluded that VC, FVC, FEV1, and FEF_25-75%_ in the exposed group were significantly lower than in the reference group. Conversely, the results of some studies are not consistent with the present study. These studies showed that pulmonary function of exposed workers was normal [31,32,46]. For instance, Bohadana et al. [32] carried out a study of a group of 127 workers with an average age of 41 years and 23 years of work experience, of whom 34% were smokers. They were exposed to beech and oak wood dust with a concentration of 13 mg/m^3^ in a non-ventilated work place. Their lung function parameters did not decrease as exposure time increased. In the study of Borm et al. [31], the exposed workers had normal FVC and FEV1 values as well. The contradictory results of various studies on the prevalence of respiratory symptoms and impaired pulmonary function parameters in exposed people may be due to variations in wood dust, climate conditions, age, work experience, dust concentration, fungal and bacterial contaminants, statistical analysis, confounding variables (controlled or not controlled), and/or the quality of wood dust (hard, soft, allergenic or non-allergenic).

According to the Occupational Safety and Health Administration, a 5% drop in FEV1 during a work shift is clinically significant [47]. In this study, 35% of individuals experienced a 5% drop in FEV1. This calls for increased surveillance to identify sensitive individuals.

Consistent with previous studies [7,17,48], the dominant pattern of lung function disorders in this study was restrictive. According to some studies, sawmill workers may develop restrictive pulmonary dysfunction, which might be explained by an immunopathological reaction to intense mold exposure [49].

The results of this study showed that exposure to wood dust was associated with an increased prevalence of respiratory symptoms, as well as both acute (i.e., partially reversible) and chronic (i.e., irreversible) decrements in some lung function parameters. In this study, the dominant pattern of pulmonary disorders was restrictive. The number of smokers and the number of cigarettes smoked per day did not differ significantly between the exposed and reference groups. Additionally, after adjusting for potential confounders, significant associations remained between exposure to wood dust and decreased PFT values and increased prevalence of respiratory symptoms.

Therefore, the findings of this study can be considered independent of smoking and could be attributed to exposure, although causeand-effect relationships cannot be established from cross-sectional studies, such as the present investigation.

Engineering measures, such as proper local exhaust ventilation systems, administrative measures (reducing daily exposure to wood dust), appropriate respiratory protective equipment, proper training, and changes in cleaning procedures are required to prevent acute and chronic pulmonary diseases among sawmill workers.

## Figures and Tables

**Table 1.** Demographic variables, cigarette smoking and level of exposure to bioaerosol particles

Variables	Exposed	Non-exposed	p-value
Age (yr)	37.41±12.82	40.42±10.53	0.07^1^
Work experience (yr)	13.60±9.91	16.09±9.53	0.07^1^
Height (cm)	172.98±7.33	173.60±7.47	0.55^1^
Weight (kg)	72.52±13.00	75.50±10.23	0.07^1^
Duration of smoking (yr)	7.93±9.12	7.25±12.56	0.66^1^
No. of cigarettes smoked per day	5.89±9.02	5.77±8.68	0.92^1^
Smokers (%)	41.0	38.0	0.66^2^
Marital status (n)			0.74^2^
Single	23	26	
Married	77	74	
Educational level (n)			<0.001^2^
High school diploma	95	64	
Higher than high school diploma	5	36	
Concentration of respirable dust (mg/m^3^)	6.76±1.71	-	-
Concentration of inhalable dust (mg/m^3^)	2.44±0.66	-	-
Bacterial concentration (CFU/m^3^)	756.38±285.55	-	-
Fungal concentration (CFU/m^3^)	299.15±239.15	-	-

Values are presented as mean±standard deviation. CFU, colony-forming unit.

1 t-test.

2 Chi-square or Fisher exact test.

**Table 2.** Frequency of respiratory symptoms in the exposed and non-exposed groups

Symptoms	Exposed (%)	Non-ex- posed (%)	Odds ratio	p-value^1^
Wheezing	37	11	4.80	<0.001
Chest tightness	27	13	2.47	0.01
Cough	28	3	12.57	<0.001
Chronic cough	15	6	2.76	0.04
Phlegm	24	6	4.94	<0.001
Chronic phlegm	14	8	2.02	0.17
Dyspnea	74	38	4.64	<0.001

1 Chi-square or Fisher exact test.

**Table 3.** The results of spirometry for exposed and non-exposed workers

Variables	Non-exposed (n=100)	Exposed (n=100)		p-value		
		Pre-shift	Post-shift	Pre-shift vs. post-shift^1^	Pre-shift vs. reference^2^	Post-shift vs. reference^2^
VC	90.00±12.16	81.36±12.71	75.31±13.13	<0.001	<0.001	<0.001
FVC	92.98±11.43	86.40±15.24	83.13±17.82	0.03	<0.001	<0.001
FEV1	94.50±11.63	88.34±15.14	84.89±14.07	0.008	0.002	<0.001
FEV1/FVC	83.45±6.82	85.46±8.05	85.67±9.71	0.83	0.06	0.06
FEV1/VC	84.34±14.24	86.90±14.50	89.77±16.29	0.07	0.13	0.006
PEF	85.56±20.75	78.73±19.07	76.23±16.68	0.12	0.02	0.001

Values are presented as mean±standard deviation. VC, vital capacity; FVC, forced vital capacity; FEV1, forced expiratory volume in the first second; PEF, peak expiratory flow.

1 Paired t-test.

2 Independent sample t-test.

**Table 4.** Pulmonary function test values among smokers in the exposed and control groups

Variables	Exposed (n=41)	Non-exposed (n=38)	p-value^1^
VC	74.58±12.48	90.76±14.69	<0.001
FVC	86.97±16.92	94.10±11.35	0.03
FEV1	86.31±14.37	94.55±13.68	0.01
FEV1/FVC	83.82±10.76	82.06±8.39	0.42
FEV1/VC	91.82±17.49	84.78±19.77	0.10
PEF	76.46±15.98	85.76±20.76	0.03

Values are presented as mean±standard deviation. VC, vital capacity; FVC, forced vital capacity; FEV1, forced expiratory volume in the first second; PEF, peak expiratory flow.

1 Independent sample t-test.

**Table 5.** Association between exposure to wood dust and symptoms of respiratory disease

Variables	OR (95% CI)	p-value^1^
Wheezing	4.80 (2.26, 10.21)	<0.001
Chest tightness	2.52 (1.19, 5.36)	0.02
Cough	12.06 (3.47, 41.93)	<0.001
Chronic cough	2.74 (1.00, 7.46)	0.05
Phlegm	4.20 (1.57, 11.25)	0.004
Chronic phlegm	1.59 (2.39, 8.11)	0.34
Dyspnea	4.40 (2.39, 8.11)	<0.001

OR, odds ratio; CI, confidence interval.

1 Logistic regression.

**Table 6.** Association between exposure to wood dust and changes in pulmonary function tests

Variables	β (95% CI)	p-value^1^
VC	-14.69 (-18.22, -11.16)	<0.001
FVC	-10.38 (-14.67, -6.09)	<0.001
FEV1	-10.55 (-14.28, -6.81)	<0.001
FEV1/FVC	-0.14 (-0.24, -0.04)	0.004
FEV1/VC	5.53 (1.25, 9.80)	0.01
PEF	-8.50 (-13.73, -3.27)	<0.001

CI, confidence interval; VC, vital capacity; FVC, forced vital capacity; FEV1, forced expiratory volume in the first second; PEF, peak expiratory flow.

1 Multiple linear regression analysis.
